# Cortical plasticity within and across lifetimes: how can development inform us about phenotypic transformations?

**DOI:** 10.3389/fnhum.2013.00620

**Published:** 2013-10-09

**Authors:** Leah Krubitzer, James C. Dooley

**Affiliations:** ^1^Center for Neuroscience, University of CaliforniaDavis, Davis, CA, USA; ^2^Department of Psychology, University of CaliforniaDavis, Davis, CA, USA

**Keywords:** epigenetic, comparative neuroanatomy, cortical development, evolution, Evo-Devo

## Abstract

The neocortex is the part of the mammalian brain that is involved in perception, cognition, and volitional motor control. It is a highly dynamic structure that is dramatically altered within the lifetime of an animal and in different lineages throughout the course of evolution. These alterations account for the remarkable variations in behavior that species exhibit. Of particular interest is how these cortical phenotypes change within the lifetime of the individual and eventually evolve in species over time. Because we cannot study the evolution of the neocortex directly we use comparative analysis to appreciate the types of changes that have been made to the neocortex and the similarities that exist across taxa. Developmental studies inform us about how these phenotypic transitions may arise by alterations in developmental cascades or changes in the physical environment in which the brain develops. Both genes and the sensory environment contribute to aspects of the phenotype and similar features, such as the size of a cortical field, can be altered in a variety of ways. Although both genes and the laws of physics place constraints on the evolution of the neocortex, mammals have evolved a number of mechanisms that allow them to loosen these constraints and often alter the course of their own evolution.

“We certainly need to remember that between the genotype and phenotype, and connecting them to each other, there lies a whole complex of developmental processes.”

Waddington, 1942.

Evolution and development of the nervous system are inextricably intertwined. Studies that link these two biological processes have recently re-emerged from the older foundations of comparative neuroanatomy and descriptive neurodevelopment as the flashy new discipline often referred to as “Evo-Devo.” This re-awakening was made possible by two events. First, descriptive neurodevelopment transformed into an experimental discipline with the advent of molecular and genetic techniques that allowed scientists to figuratively “poke the frog.” The ability to make targeted changes, via genetic manipulations that differentially affect specific aspects of development, allowed us to appreciate the contingencies inherent in the developmental process and to understand the role these genetic cascades play in the construction of specific features of the nervous system. Importantly, it is becoming increasingly clear that the cortical field is not a static entity, but transforms continually at all stages of development. The second event was the emergence of new technologies in genetics that allowed scientists to decode and compare entire genomes of selected species. The prospect that this would ultimately uncover the fundamental differences between species propelled the somewhat aging field of evolutionary neurobiology to the forefront of neuroscience.

Our laboratory has long been interested in the evolution of the neocortex and has used comparative studies to formulate testable hypotheses regarding neurodevelopment. Specifically we are interested in the developmental mechanisms that give rise to aspects of neocortical organization that have changed significantly in species over the course of evolution. We focus on the neocortex for two important reasons. The first is that the neocortex is the portion of the brain involved in complex behaviors including perception, cognition, language, and temporal planning of events. Second, it is the portion of the brain that has changed most dramatically in mammals compared to other parts of the brain (Krubitzer, 2007). The neocortex has expanded tremendously in human and non-human primates, and has expanded independently in several other orders of mammals including cetaceans, proboscidea, and rodentia. However, it is not just an increase in the size that distinguishes some large-brained mammals from others, but also an increase in the number of functional subdivisions, and importantly, alterations in their patterns of connectivity. Studies of endocasts of the skulls of early mammals (Luo et al., 2001) as well as comparative studies (Meredith et al., 2011; O'Leary et al., 2013) suggest that the first mammals that roamed the earth some 200 million years ago had a small neocortex with perhaps 10–15 cortical fields, and a relatively large pyriform cortex and olfactory bulbs (Rowe et al., 2011; Dooley et al., 2013; see Kaas, 2011 for review). This early mammaliform and its descendants evolved to produce some extant species with a neocortex that dominates the rest of the nervous system and contains billions of cells with hundreds of cortical fields. The question is how did this occur, and what factors contribute to this increased complexity of form, function and behavior.

One obstacle in addressing this question is that cortical evolution in mammals cannot be studied directly. The types of changes that brains have evolved occur over multiple generations and often take tens of thousands to millions of years to emerge. However, there are two ways to circumvent this problem. The first is to examine the products of evolution, extant animal brains and bodies, to determine *what* changes have occurred. This comparative approach has been used to good effect to appreciate common features of the neocortex that all species share as well as derivations that have been made to the basic plan of organization. Unfortunately, comparative studies do not provide information on how phenotypic transformations occur, or the rate at which changes can happen. To appreciate how changes occurred we study the developmental mechanisms that are proposed to give rise to some aspect of cortical organization. Thus, it is critical to appreciate how processes such as neurogenesis, cell migration, neuronal differentiation, and axon guidance are altered in mammals with different cortical phenotypes. These alterations give rise to some feature of organization that we study in our comparative analysis such as cortical sheet size, cortical field size, and connectivity. For these reasons developmental studies tell us *how* phenotypic changes occur.

It is important to stress that any theory of brain evolution, cortical function or cortical plasticity should not consider the neocortex in isolation, but must recognize that the neocortex is only one component of the entire nervous system. Further, the nervous system is embedded in a body, which interacts with other organisms and the environment. This group of organisms and their environment generates a complex and highly dynamic “collective biomass” that itself has emergent properties which differ from, and in some instances exceed, the individual elements of which it is composed (Krubitzer, 2009). Further, it is critical to appreciate that the relationship between genes, the brain, the body, and the target of natural selection (behavior) is often highly convoluted and indirect (see Krubitzer and Seelke, 2012 for review).

In the following review we first provide an overview from comparative studies that outlines common features of cortical organization that have been identified in all species examined and how aspects of this common plan have been modified. Second, we address the question of how these phenotypic transformations have occurred, including a review of studies that examine how genes contribute to neurogenesis, cortical sheet size, and aspects of cortical arealization across development. We underscore the importance of examining not only genes intrinsic to the neocortex, but also genes that regulate the body plan and limb and effector morphology. Next, we discuss activity-driven alterations to the cortical phenotype. To appreciate the gene/environment interactions we look to natural examples of extreme morphological/behavioral specialization that is accompanied by exaggerated aspects of cortical organization, and describe our developmental studies in which we try to mimic these changes to the neocortex by radically altering sensory inputs. Finally, we describe more subtle examples in which animals of the same species, reared in different sensory environments, develop alterations to the cortical phenotype. We discuss potential epigenetic mechanisms that construct context dependent alterations to the phenotype.

## What is the plan and how has it changed?

Comparative studies use multiple criteria to define a cortical field including functional techniques (e.g., electrophysiological recording, imaging, intracortical microstimulation), combined with architectonic and neuroanatomical techniques. In our experiments we survey a large extent of the neocortex by recording neural activity from hundreds of sites while successively presenting visual, auditory, and tactile stimulation to determine sensory domain allocation (the amount of cortex devoted to a particular sensory system). These techniques also allow us to determine the number and overall organization of different cortical fields within a sensory domain. These data can be combined with architectonic techniques in which the region of interest is stained for particular cell types, myelinated axons, enzymatic activity, or any number of other histochemical markers that illuminate cortical field boundaries, which are then directly related to functional techniques. Cortical regions can also be divided using neuroanatomical techniques to examine subcortical, cortical and interhemispheric connections of the field in question.

**Figure 1:**
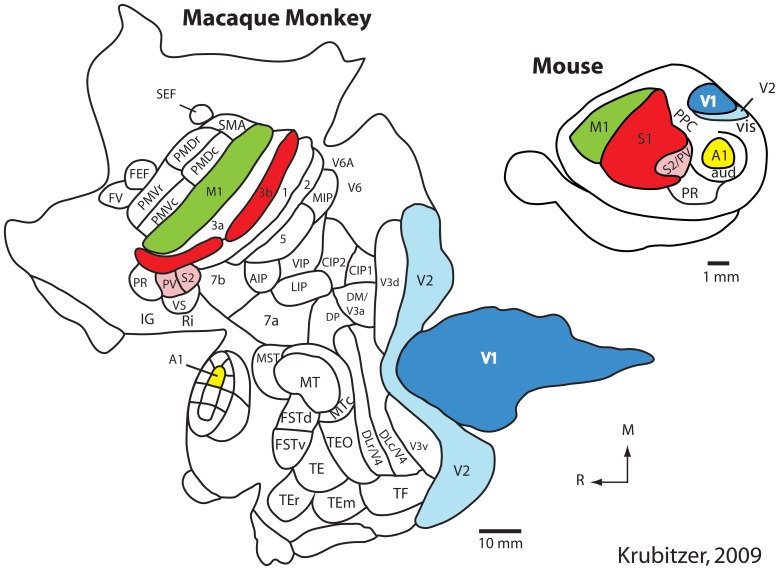
**The organization of the neocortex in the macaque monkey and mouse in cortex that has been peeled from the brainstem and thalamus and flattened.** Homologous cortical fields include the primary somatosensory area (S1/3b; red), the second somatosensory area and the parietal ventral area (S2/PV; rose), the primary visual area (V1; dark blue), the second visual area (V2; light blue), the primary auditory area (A1; yellow) and motor cortex (M1; green). While this common plan of organization can be identified in these species, there are also notable differences. Specifically, in macaque monkeys the neocortex has greatly expanded and multiple additional cortical areas have been added. Further, the relative size of homologous cortical fields (as a percentage of overall cortical area) is different. While different investigators have proposed different schemes of cortical organization in the macaque and mouse, it is clear that macaque monkeys have many more cortical fields than does the mouse. Modified from Krubitzer (2009). See Table 1 for abbreviations. All abbreviations for the macaque monkey are not provided; this figure simply demonstrates that the number of cortical fields has increased.

Using such techniques in a number of different species, our own and other laboratories have generated schemes of cortical organization composed of architectonically, connectionally, and functionally distinct maps of the sensory receptor arrays associated with visual, somatosensory, and auditory processing. These comparative studies indicate that there is a constellation of cortical fields that all mammals possess that can be defined using multiple criteria. These include primary visual, somatosensory, and auditory cortical fields (V1, S1, and A1 respectively) as well as one or two additional sensory areas (Figure 1; e.g., V2, S2/PV, R) (Dooley et al., 2013; see Kaas, 2011 for review).

**Table 1:** **Abbreviations used throughout the text**.

A1—Primary auditory cortex
Emx2—Empty spiracles homeobox 2—transcription factor expressed in a caudal (high) rostral (low) gradient
FGF8—Fibroblast growth factor 8—morphogen important for generating the rostral-caudal axis
GR—Glucocorticoid receptor
HPA—Hypothalamic–pituitary–adrenal
IPC—Intermediate progenitor cells
ISVZ—Inner subventricular zone
LG—Licking and grooming
M1—Primary motor cortex
oRG—Outer radial glial cells
OSVZ—Outer subventricular zone
Pax6—Paired box protein 6—transcription factor expressed in a rostral (high) caudal (low) gradient
PV—Parietal ventral area
R—Rostral somatosensory field
RG—Radial glial cells
S1—Primary somatosensory cortex
S2—Second somatosensory area
SVZ—Subventricular zone
V1—Primary visual cortex
V2—Second visual area
VZ—Ventricular zone

Interestingly, these fields are present even in the absence of apparent use in animals showing extreme specialization such as blind mole rats (Cooper et al., 1993; Bronchti et al., 2002). Moreover, independently evolved modifications to this plan take a similar form in different lineages (Krubitzer and Kaas, 2005). Systems-level evolved changes in cortical organization include:

1. The absolute and relative size of the cortical sheet
2. Sensory domain allocation
3. Response properties and stimulus preference of neurons within a cortical field
4. Relative size of cortical fields
5. Magnification of behaviorally relevant body parts
6. Addition of modules to cortical fields
7. Number of cortical fields
8. Connections of cortical fields

The persistence of the shared cortical field plan across all mammals and the similarities in its modifications suggest that there are large constraints on how cortical fields evolve. For further discussion of constraints and variability see Krubitzer and Seelke, 2012.

## What factors contribute to these changes?

As noted above, while comparative studies allow us to appreciate the types of changes that have been made to the neocortex, developmental studies provide insights into how these changes occur. Thus, the next question that arises from our comparative analysis is what factors contribute to within-species variability of the features of cortical organization listed above. This is a question that has been posed for decades, commonly presented as a nature vs. nurture debate. Recently, advances in comparative genomics and epigenetics confirm the contributions of both genetic and context-dependent factors to different aspects of the cortical phenotype and within-species variability. Still contentious, however, is the extent to which each factor shapes or constructs any given phenotype.

Traditionally, context-dependent changes to cell phenotypes during development had been referred to as “epigenetic” (Waddington, 1942). Waddington coined the term epigenetics to explain how cells in the developing organism can have the same genotype, but gradually differentiate into different tissue. This phenomenon underscores that there is not a one-to-one correspondence between genotype and phenotype, and that there must be something beyond the genotype that generates this diversity. We now appreciate that this same ability to alter a cellular, systems, or behavioral phenotype occurs in mature, non-dividing cells in the central nervous system (Day and Sweatt, 2010), and this phenomenon has also been termed epigenetics. While early in development, context-dependent changes may be as simple as folic acid availability or location of a particular cell on a developing blastocyst, as development progresses, the context (and thus its potential for change) becomes more complicated. This is particularly true for the mammalian neocortex, where environmental context routinely molds the phenotype. A particular cortical phenotype may persist for multiple generations if the context in which it develops is static, but these features of cortical organization are not inherited and thus do not evolve. However, new studies, which we will discuss below, have overturned some assumptions about heritability and have begun to uncover the mechanisms that generate contextually dependent phenotypes that can be expressed in multiple generations, and in some instances become incorporated into the germ line and evolve.

### Cortical sheet size

**Figure 2:**
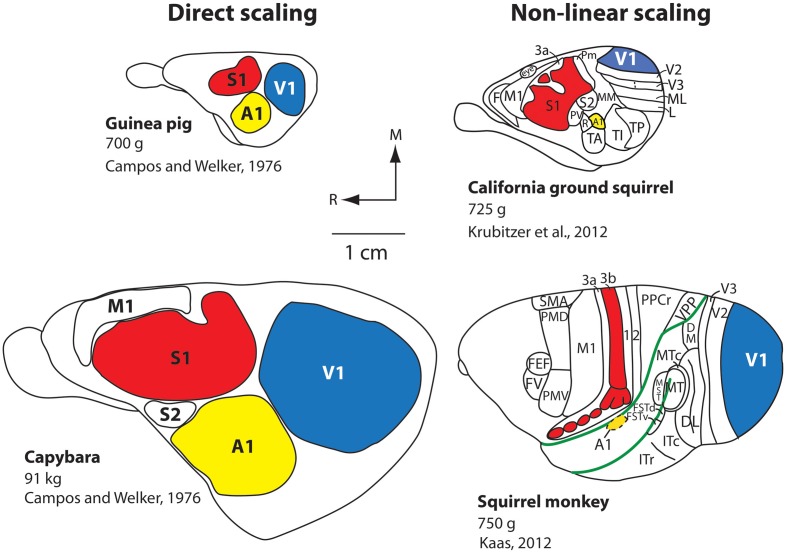
**Scaling of the neocortex in different mammals.** Comparative studies demonstrate that the neocortex scales linearly or non-linearly. Capybaras can weigh up to 91 kg and have an enlarged brain and neocortex compared to the closely related guinea pig, which weighs 700 g. The more distantly related California ground squirrel has a similar body size to that of the guinea pig, but the scaling of the cortical sheet and cortical fields compared to the capybara is non-linear, and there is an increase in the number of cortical fields. An extreme example of a non-linear increase in the size of the cortical sheet is observed in squirrel monkeys. Although squirrel monkeys are of a similar weight (750 g) compared to the guinea pig and California ground squirrel, they have a relatively large neocortex (about the size of the capybara's), a relative decrease in the size of primary cortical fields (e.g., A1, S1, V1) as a percentage of overall cortical area, and the addition of cortical fields (note that not all known cortical fields in the squirrel monkey neocortex are shown; the blank areas contain additional cortical fields). All brains are drawn to scale. The work on the guinea pig and capybara is modified from Campos and Welker (1976); the divisions of the ground squirrel are redrawn from Krubitzer et al. (2011); divisions of the squirrel monkey are redrawn from Kaas (2012). Other conventions as in previous figure.

One of the well-defined systems-level changes to the brain has been an expansion of the cortical sheet. Throughout the course of mammalian evolution, this expansion has taken two different forms: (1) Absolute increase in size (direct scaling), and (2) Relative increases in size (non-linear scaling). Direct scaling consistently occurs with an increase in body size. The brain scales directly with the body, and every structure, including the neocortex and constituent fields, expand roughly equally. This is exemplified by the comparison of two closely-related rodents: The guinea pig (700 g) and the South American capybara, the largest rodent on earth which weighs up to 91 kg (200 lbs; Figure 2). The neocortex of the guinea pig is much smaller than that of the capybara, but relative to body size, the size of the neocortex and the primary sensory fields are comparable (Campos and Welker, 1976).

The second type of increase in the size of the cortical sheet is non-linear and is related to a different type of cortical organization. The California ground squirrel is similar in overall body size to the guinea pig (700 g), but its neocortex is substantially larger both absolutely and relative to the body or rest of the brain (Campi and Krubitzer, 2010). This non-linear increase in the size of the cortical sheet is accompanied by a decrease in the overall percentage of neocortex occupied by primary sensory areas along with an increase in the absolute number of cortical fields on the cortical sheet (Figure 2), a pattern even better exemplified in non-human primates such as squirrel monkeys. Squirrel monkeys have about the same body mass as both California ground squirrels and guinea pigs (750 g), but have an extraordinarily large neocortex compared to the body and the rest of the brain, and a dramatic increase in the number of cortical fields. Thus, an absolute (linear) increase in the size of the neocortex is not sufficient to yield an increase in its complexity (i.e., more cortical fields/changes in connections). Conversely, a relative (non-linear) increase in the size of the neocortex does appear to be necessary to increase number of cortical fields, but may not be sufficient to induce this change.

Some questions that emerge are: (1) How is an increase in the size of the cortical sheet accomplished? (2) Are the underlying mechanisms of direct and non-linear scaling of the cortex different? (3) What is the link between changes in brain and body size? and (4) Are the underlying mechanisms that give rise to increases in cortical sheet size similar in species that have independently increased the size of the cortical sheet (e.g., primates and cetaceans)?

### The evolution of neurogenesis

**Figure 3:**
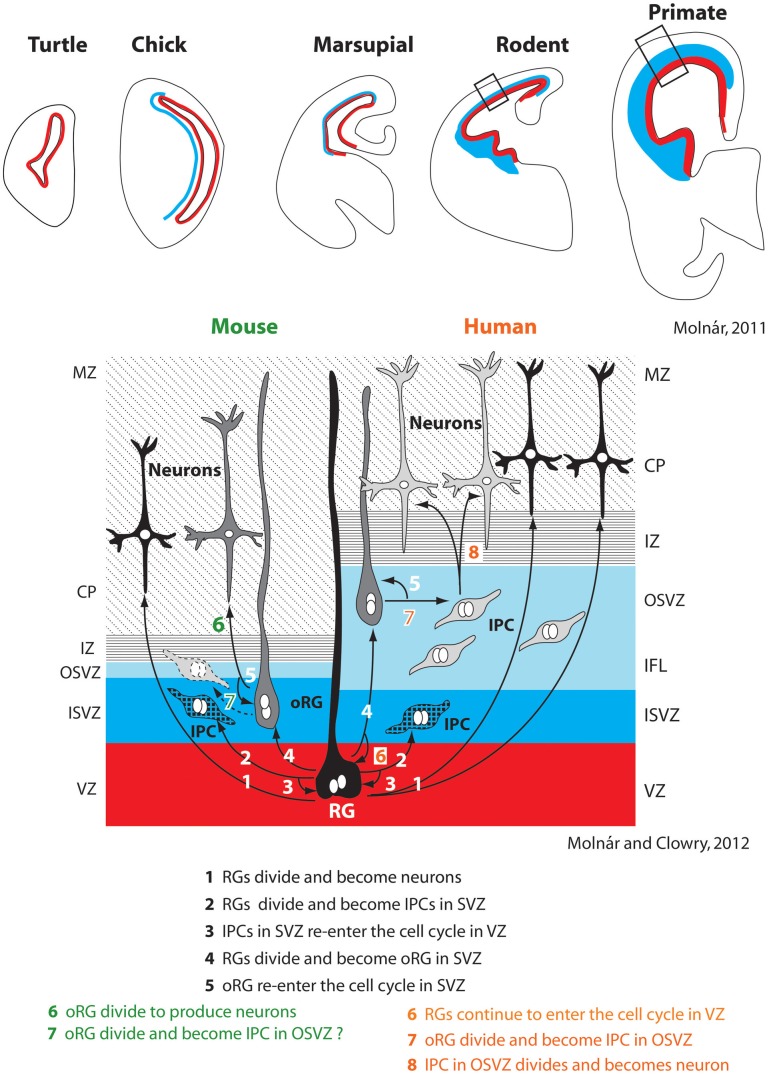
**Changes in the subventricular zone (SVZ; blue) in vertebrates (top row).** Specific alterations in cell cycle kinetics, the overall thickness of the SVZ, the proportion of the SVZ corresponding to the inner and outer layers (ISVZ and OSVZ respectively), and the proportion of asymmetrical radial glial (RG) and outer radial glial (oRG) cell divisions producing intermediate progenitor cells account for expansion of the neocortex in some lineages, such as primates (right half of bottom panel). The SVZ and particularly the OSVZ is larger in primates than in many other species. While mammals with both a large and small neocortex share a number of aspects of neurogenesis (steps 1–5 bottom figure), several additional adaptations are observed in animals with a large neocortex such as primates. This includes increased thickness of particular layers (such as the OSVZ), additional rounds of division for RGs that re-enter the cell cycle in the ventricular zone (VZ) (6), division of oRGs into intermediate progenitor cells (IPC) in the OSVZ (7), which ultimately divide again to produce neurons (8). Whether oRGs also divide to produce IPCs in rodents is still contentious (dotted line on left side, 7). These figures have been modified from Molnár (2011) and Molnár and Molnár and Clowry (2012). Abbreviations in Table 1.

Recent studies of neurogenesis have made important inroads into understanding, at least in some species, the mechanisms that contribute to tangential increases in the size of cortical sheet during development and how these mechanisms may be altered in different species to produce differences in the size of the cortical sheet. Historically, researchers investigating neurogenesis have hypothesized that animals with larger (usually gyrencephalic) brains have an increased duration of neurogenesis and modified cell cycle kinetics, and that such alterations are not present in small (lissencephalic) brained animals. This notion is supported by comparative studies in mice and macaque monkeys that demonstrate that more rounds of cell division occur over a longer period of time in the macaque compared to the mouse (Takahashi et al., 1995; Kornack and Rakic, 1998; Kornack, 2000). Subsequent studies described additional changes in neurogenesis that could account for an expanded cortical sheet in some lineages. One important alteration, first described in the macaque monkey, was the presence of an outer subventricular zone (OSVZ, Smart et al., 2002; Figure 3). Additionally, within the OSVZ are proliferative radial glia-like cells termed outer radial glial cells (oRG) that generate neurons that will compose the cerebral cortex (Fietz et al., 2010; Hansen et al., 2010; Reillo et al., 2011; Shitamukai et al., 2011; Wang et al., 2011; Martínez-Cerdeño et al., 2012). This large OSVZ and the oRG proliferative cells, at least in part, account for the exponential expansion of the cerebral cortex in some orders such as primates.

Initially, this expanded OSVZ and the corresponding oRG cells were considered an adaptation limited to large-brained, gyrencephalic mammals, but recently a much smaller OSVZ has been described in rats. This OSVZ shares many of the same features found in larger brained ferrets and macaques, such as the presence of oRG proliferative cells (Martínez-Cerdeño et al., 2012). There is also evidence for an OSVZ in the marmoset (a dwarfed, nearly lissencephalic primate) and the agouti (a gyrencephalic rodent; García-Moreno et al., 2012). Thus, small-brained mammals from multiple orders possess the basic ventricular compartments (OSVZ) and proliferative cells (oRG) that can generate expansions in the cortical sheet.

If not the presence of OSVZ and oRG, what differentiates large and small brains? It appears that large-brained animals have an increased generation of intermediate progenitor cells (Wang et al., 2011), a greater number of oRGs present across development (Hevner and Haydar, 2012), and a thicker OSVZ (Bystron et al., 2008; Martínez-Cerdeño et al., 2012), all of which can lead to a larger neocortex with a greater number of neurons (Figure 3). Additionally, while oRGs have been shown to produce neurons and intermediate progenitor cells in primates, which further divide into post-mitotic neurons (Hansen et al., 2010), in mice they have only been shown to divide directly into neurons (Wang et al., 2011), although this is not the case for all rodents (Martínez-Cerdeño et al., 2012). While more comparative studies need to be done, mounting evidence suggests that changes in the size of the cortical sheet are due to expansions of existing populations of cells and cell cycle kinetics, rather than the creation of novel mechanisms.

Exciting research published in the last year has identified proteins which appear to regulate the population of oRG cells (Trnp1; Stahl et al., 2013) and intermediate progenitor cells (BAF170; Tuoc et al., 2013), such that both over expression and under expression of these proteins in the neocortex alter the number of these progenitor cells and ultimately alter cortical sheet size. Further, research by Nonaka-Kinoshita et al. (2013) shows that increasing the pool of basal progenitors in the lissencephalic mouse increases the size of the neocortical sheet, but is not sufficient to induce gyrencephally; however, the same manipulation in the naturally gyrecephalic ferret both increased the size of the cortical sheet and induced additional cortical sulci. Thus, while increasing the number of progenitor cells invariably leads to a larger cortical sheet, existing data suggests that a sufficient population of oRG cells must also be present to create sulci and gyri in a naturally lissencephalic cortex (Stahl et al., 2013; Tuoc et al., 2013).

It is important to note that epigenetic events can also regulate the size of the cortical sheet. These context-dependent alterations in cortical sheet size appear to be caused by a variety of factors. For example, it has been well documented that folic acid (and cholate) regulates neurogenesis and apoptosis in the developing fetal brain, and differences in intake can alter the number of progenitor cells undergoing mitosis by 33–54% in the neocortex of mice (Craciunescu et al., 2004, 2010). Although a number of studies have demonstrated that domestication also has a profound impact on the size of the cortical sheet (see Kruska, 2005), it is difficult to disambiguate the contribution of genes vs. environment on cortical sheet size.

### Cortical field size and connectivity

**Figure 4:**
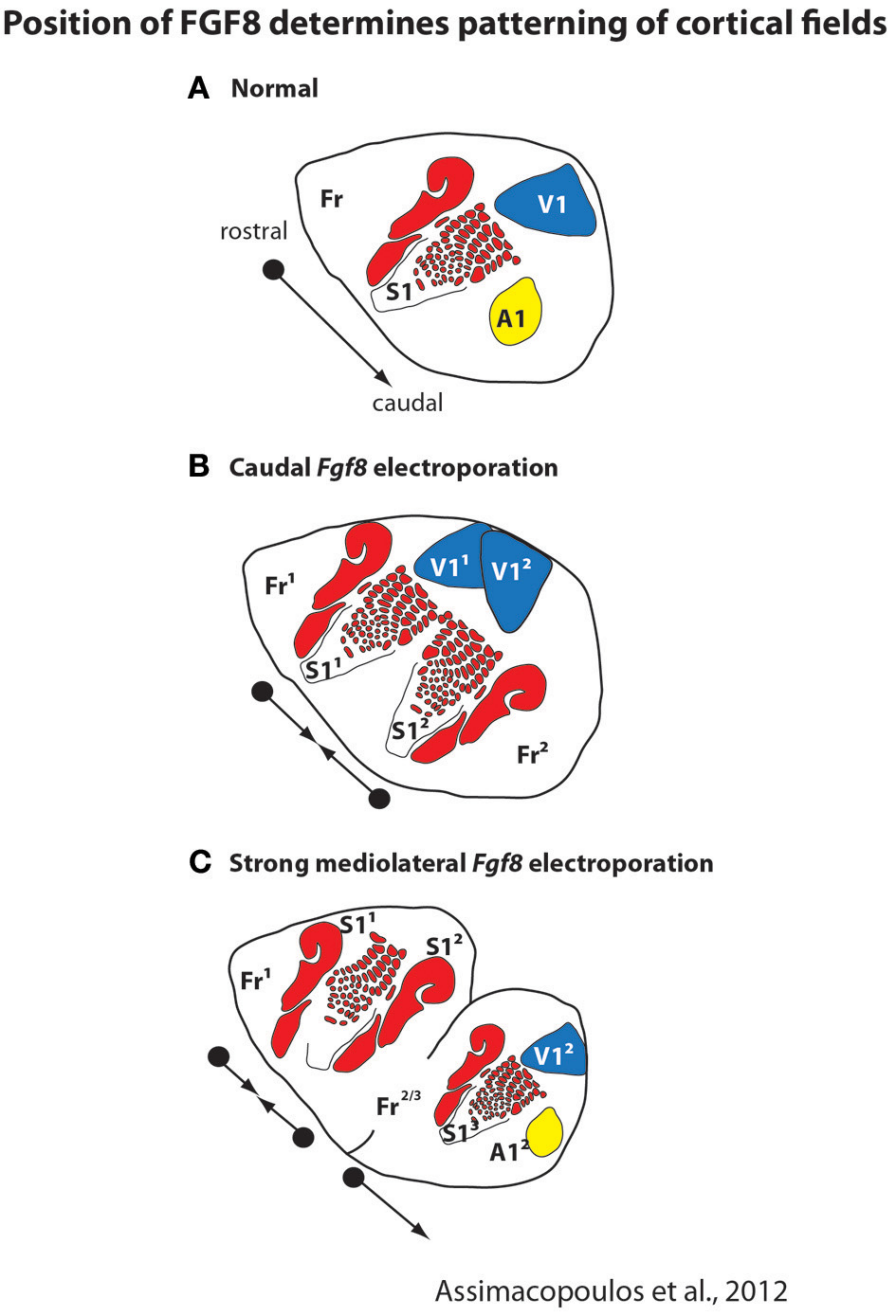
**Early in development the position and strength of morphogens, such as FGF8, determines the location and patterning of cortical fields on the cortical sheet.** In normal mice **(A)**, FGF8 is expressed early in development in the rostromedial neocortical primordium and forms a rostro-caudal gradient that regulates subsequent rostrocaudal patterns of gene expression. Studies in which *Fgf8* is electroporated at differing levels and in different locations in the embryonic mouse (E10.5) demonstrate its importance as an early cortical map organizer. Ectopic placement can result in the duplication of a cortical field (**B**; S1^1^ and S1^2^) or multiple cortical fields arranged along variant rostro-caudal axes **(C)**. These new duplicated fields are also functionally distinct and form topographic maps, as in normal animals. Modified from Assimacopoulos et al. (2012).

Like cortical sheet size, both genetic and epigenetic factors contribute to aspects of cortical field size and connectivity. A plethora of studies demonstrate that intrinsic factors contribute to a number of features of cortical organization including relative position on the cortical sheet, relative size of the cortical field, and cortical field connections (e.g., Bishop et al., 2000; O'Leary and Sahara, 2008; Assimacopoulos et al., 2012). For example, ground-breaking studies from a number of laboratories demonstrated that morphogens such as FGF8 generate a rostral cortical identity, and that these early signaling centers set up genetic cascades which regulate position, size, and connectivity of cortical fields. The importance of these early signaling centers is clearly demonstrated in recent studies in which *Fgf8* was electroporated into different regions of the developing mouse embryo and duplicate fields (with rostrocaudal axes) were observed (Assimacopoulos et al., 2012, Figure 4). Electroporating *Fgf8* into a caudal (aberrant) location results in an almost complete duplication of cortical maps with a mirror reversal of V1 and S1 at mid cortex, with two distinct “rostral” poles and a shared caudal pole boundary (Figure 4B). This compelling result presents a possible mechanism for mirror reversal organization of cortical fields (such as in anterior parietal fields 3a, 3b, 1 and 2 in primates). These naturally occurring duplicate somatotopic maps could have originated as an alteration in location and strength of these early signaling centers in parietal cortex. While the connectivity of duplicated cortical maps is not known, they do appear to be functionally responsive and topographically organized (Assimacopoulos et al., 2012).

**Figure 5:**
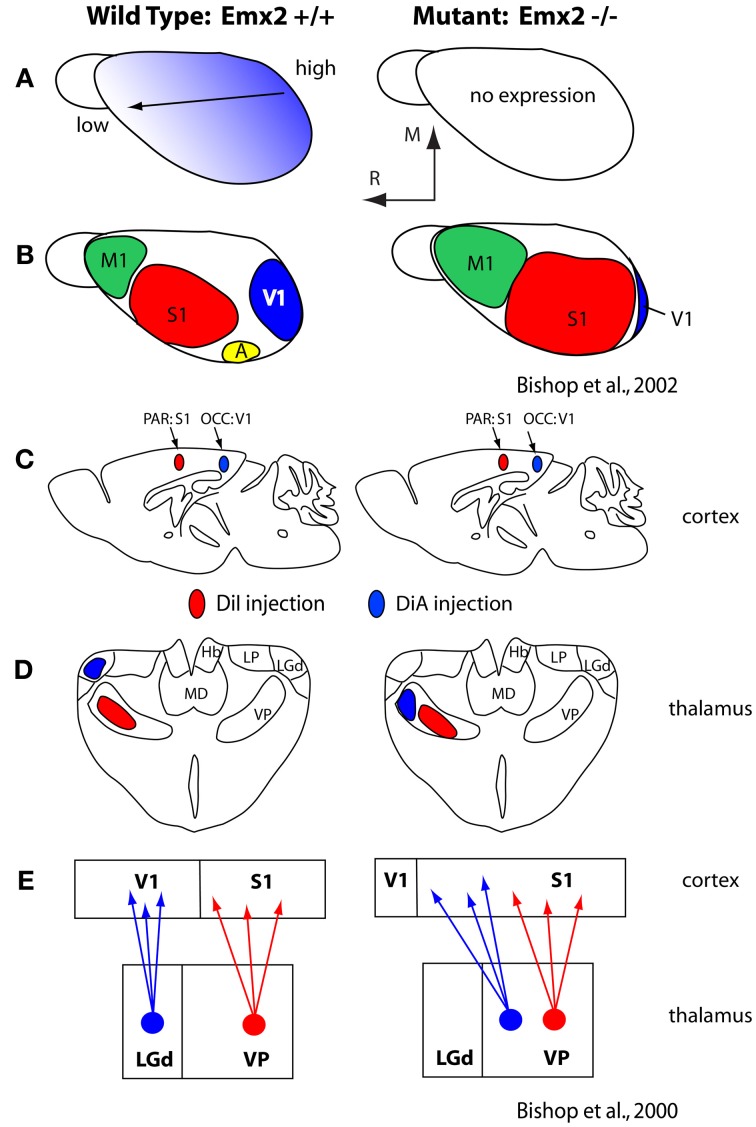
**(A)** Graded expression of *Emx2* in the normal (left) and mutant (right) mice. Normal expression generates normal patterns of cortical fields (**B**; left) and absence of *Emx2* generates altered patterns of organization such that caudal domains have decreased (V1 is small) and rostral domains have expanded into the caudal territories (**B**; right). **(C)** Injections into parietal cortex (what will become S1) and occipital cortex (what will become V1) demonstrated altered patterns of thalamocortical connections **(D)**. In mutants what would normally develop into visual cortex has projections from VP, normally associated with somatosensory processing. The schematic in **(E)** demonstrates normal thalamocortical connections of S1 and V1 (left) and the caudal shift of VP projections into what would normally be visual cortex (**E**; right). These figures are modified from Bishop et al. (2000, 2002). Conventions as in previous figures.

Generation of this rostral-caudal axis by FGF8 influences downstream transcription factors expressed early in development, such as *Pax6* and *Emx2*, which themselves appear to be critical for establishing appropriate expression patterns of cell adhesion molecules (Bishop et al., 2000, 2002; Hamasaki et al., 2004; O'Leary and Sahara, 2008; Figure 5). These molecules in turn regulate a number of aspects of the cortical phenotype including the relative size of cortical fields and their connectivity (Suzuki et al., 1997; Inoue et al., 1998; Bishop et al., 2002; Terakawa et al., 2013). For example, over or under expression of *Emx2* in the early developing mouse neocortex has been shown to alter expression of cell adhesion molecules (Stoykova et al., 1997; Bishop et al., 2000, 2002; Andrews and Mastick, 2003), ultimately resulting in an increase or decrease (respectively) in the size of cortical fields on the caudal pole of the neocortex including V1, and alterations in thalamocortical connections (Bishop et al., 2000; Hamasaki et al., 2004; Figure 5). Importantly, in the absence of thalamocortical afferents, the expression patterns of some of these early transcription factors and genes are maintained (Nakagawa et al., 1999) indicating that activity is not requisite for their expression and thus certain aspects of cortical organization are immutable, regardless of context.

### Genes extrinsic to the developing neocortex contribute to cortical organization and connectivity

**Figure 6:**
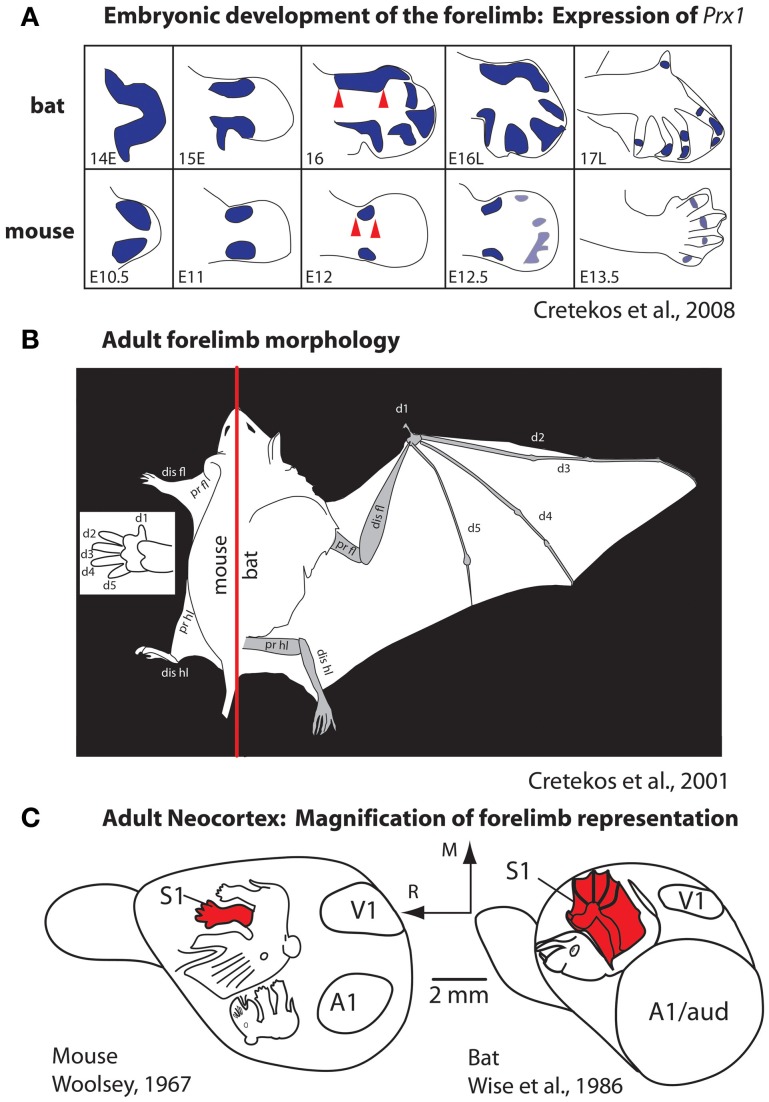
**Development (A) and morphology (B) of the forelimb, and the representation of the forelimb in somatosensory cortex (C) in mice and bats. (A)** At middle stages of forelimb development the expression of *Prx1* (purple) is expanded in the distal forelimb (red arrows). This alteration, among a number of other molecular changes, accounts for the radical differences in the rat forepaw compared to the bat wing **(B)**. These morphological differences in the distal forelimb in addition to differential use of the paw vs. wing have likely contributed to the differences in size and internal organization of the forelimb representation in S1 **(C)**. Figures are modified from Cretekos et al. (2001, 2008); Woolsey (1967), and Wise et al. (1986). Other conventions as in previous figures.

Most studies of cortical development focus almost exclusively on genes that are intrinsic to the developing neocortex. However, as noted in our introduction, brains do not develop or evolve in isolation, but in the context of the body, behavior, and a rich sensory environment generated by biological and non-biological sources. An excellent example of the interaction between genes that regulate body morphology and the effect of this on the brain and behavior comes from comparisons of limb development in species that have radically different forelimb phenotypes, such as the mouse and the short-tailed fruit bat (Figure 6). The early development of the mouse and bat forelimb is remarkably similar. However, at mid stages of limb development, the interdigit membranes in the mouse undergo apoptosis, which results in a separation of individual digits of the forepaw (Cretekos et al., 2008). Conversely, at this stage of limb development in the bat apoptosis does not occur. In addition, in the bat there is a lengthening of the forelimb and elongation of the digit phalanges. Together these alterations generate much of the phenotypic differences in these species, which in turn are related to radical differences in the use of the forelimb. Comparative studies of gene expression during limb development indicate that there are several key genetic alterations that account for these differences. In the bat, upregulation of *Prx1* results in a lengthening of the distal forelimb (Cretekos et al., 2008; Behringer et al., 2009; Figure 6A) and a posterior shift in *Hoxd13* expression reduces some skeletal elements. In the mouse, BMPs trigger apoptosis of interdigit membranes. In the bat BMPs are inhibited by *Gremlin* thus preserving interdigit membranes and this inhibition is accompanied by an increase in FGF8 in the apical ectodermal ridge, which extends the distal growth of the forelimb. Another important distinction of the bat forelimb is the presence of touch domes. These specialized receptor assemblies are found across the wing membranes and are beautifully sensitive to small changes in air pressure (Zook and Fowler, 1986; Sterbing-D'Angelo et al., 2011).

These changes to the forelimb are associated with differential use of the limb and the ability to make fine discriminations with the wing for self propelled flight and wing to mouth feeding behavior (Sterbing-D'Angelo et al., 2011). Studies of neocortical organization of the somatosensory cortex demonstrate an expansion of the forelimb and digit (wing) representation within S1 of the bat compared to the mouse (Woolsey, 1967; Wise et al., 1986; Cretekos et al., 2001, 2008, Figure 6C). Additionally differences in the interhemispheric connections of the forelimb in bats have been observed. In both large and small-brained mammals with discrete digits, the forepaw/hand representation within S1 is almost devoid of connections across hemispheres (for review, see Innocenti, 1986). These acallosal hand/paw representations in S1 and associated anterior parietal fields are particularly discrete in species like primates that use the glabrous digits as a major effector for object exploration. In bats digits 2–4 are fused by the wing membranes and tactile stimulation of the wings is used for fine control in self-propelled flight. The wing representation in primary somatosensory cortex and associated fields receives dense callosal inputs for rapid interhemispheric communication between centers that process incoming inputs and generate fine motor control of the wing during flight (Krubitzer et al., 1998).

There are numerous other model systems demonstrating the role peripheral body morphology can have on the development of the neocortex in the scientific literature. Perhaps the most extensively studied peripheral/central system is the vibrissae and their corresponding barrels in S1 (for review, see Erzurumlu and Gaspar, 2012). While a complete discussion of the experimentally-induced plasticity of this system is beyond the scope of this review, genetic manipulations have produced mice which possess additional whisker follicles (Welker and Van der Loos, 1986) or which lack several whisker follicles (North et al., 2010). Welker and Van der Loos generated six strains of mice with differing patterns of extra vibrissae and found that regardless of the peripheral patterns of vibrissae, all extra vibrissae were represented cortically with extra barrels (Welker and Van der Loos, 1986). Likewise, mice lacking particular vibrissae also lacked the corresponding barrels in S1, as the representations of these vibrissae in associated subcortical pathways (North et al., 2010).

It should be noted that environmental factors also contribute to features of body morphology and in turn brain organization. For instance, gravitational stress can affect craniomandibular morphology including bone density (Singh et al., 2005), and diet and associated mastication behavior affects craniofacial morphology (He, 2004; Koyabu and Endo, 2009). Environmental factors such as salinity, temperature and humidity also contribute to body morphology (Johnston and Gottlieb, 1990), and even sex determination (Matsumoto et al., 2013). Together these body morphology changes could radically affect a number of aspects of behavior including self-propelled flight and feeding, which in turn could alter aspects of sensorimotor cortex organization and connectivity.

What is not understood is the extent to which these changes to peripheral morphology and use can drive fundamental changes in cortical organization, connectivity, sensory mediated discriminations, perceptions, and higher level cognitive processes, and if or how these changes to the brain become genetically encoded and evolve.

### Observations from the natural world

**Figure 7:**
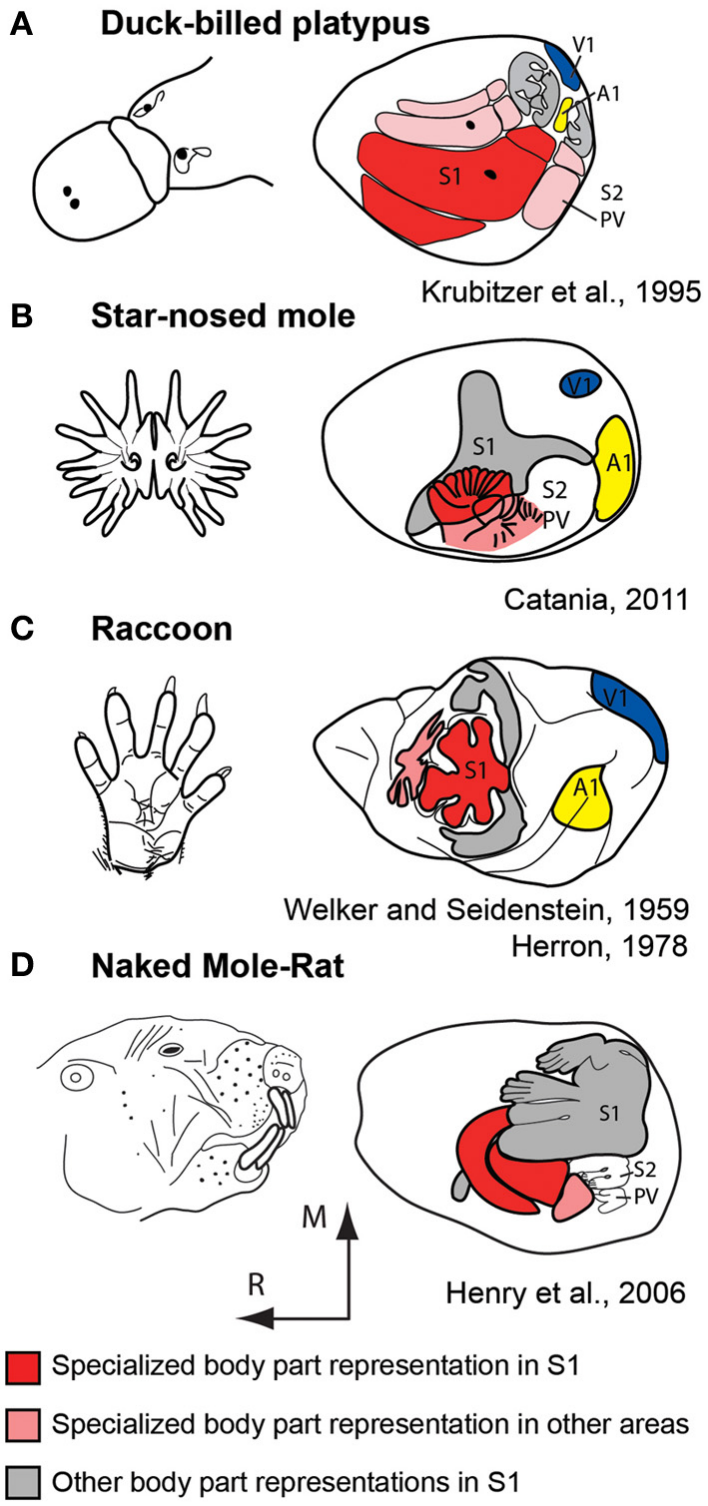
**Examples of extreme cortical magnification of behaviorally relevant effectors for somatosensory cortex of the duck-billed platypus (A), star-nosed mole (B), raccoon (C), and naked mole-rat (D).** Although the specialized morphological structure and associated sensory receptor arrays are on different body parts, the same principle of magnification in the neocortex is observed. These figures are modified from Krubitzer et al. (1995) **(A)**; Catania (2011) **(B)**; Welker and Seidenstein (1959) and Herron (1978) **(C)**; Henry et al. (2006) **(D)**. Conventions as in previous figures.

For decades comparative neurobiologists have examined mammals that have evolved extreme specializations in an attempt to uncover general rules of construction as well as constraints imposed on the evolving nervous system. These types of observations highlight features that may be more difficult to uncover when only subtle differences exist in some aspect of brain organization in different species. One of the most extraordinary examples of this comes from studies of the duck-billed platypus (Figure 7A). The platypus has evolved electrosensory receptors that form anteroposterior rows on the bill that interdigitate with rows of mechanosensory receptors (Scheich et al., 1986; Gregory et al., 1987, 1988; Iggo et al., 1992). Most activities of the platypus are performed in the water during which time its eyes, ears and nose are closed. Thus, inputs from the bill, and to a limited extent the body, are the brain's source of information about the animal's immediate environment. Examination of the organization of the neocortex indicates an enormous expansion of the bill representation with clear territories devoted to processing electrosensory vs. mechanosensory inputs. There are three separate representations of the bill, which together occupy about 50% of the cortical sheet. In S1 alone, this magnification of a behaviorally relevant body part is enormous; the bill representation occupies 95% of S1 (Krubitzer et al., 1995).

Additional examples of extreme magnification have been observed in the primary somatosensory area of a number of mammals including the naked mole rat and star-nosed mole (Figure 7). This expansion of cortical territory related to effector specific inputs and active use is also observed in other sensory systems including an expansion of central vision in diurnal primates, and an expansion of ultrasonic frequency representations in echolocating bats (Suga et al., 1987). These alterations are due to changes in peripheral morphology, use, and the physical environment in which the animal develops and ultimately lives.

**Figure 8:**
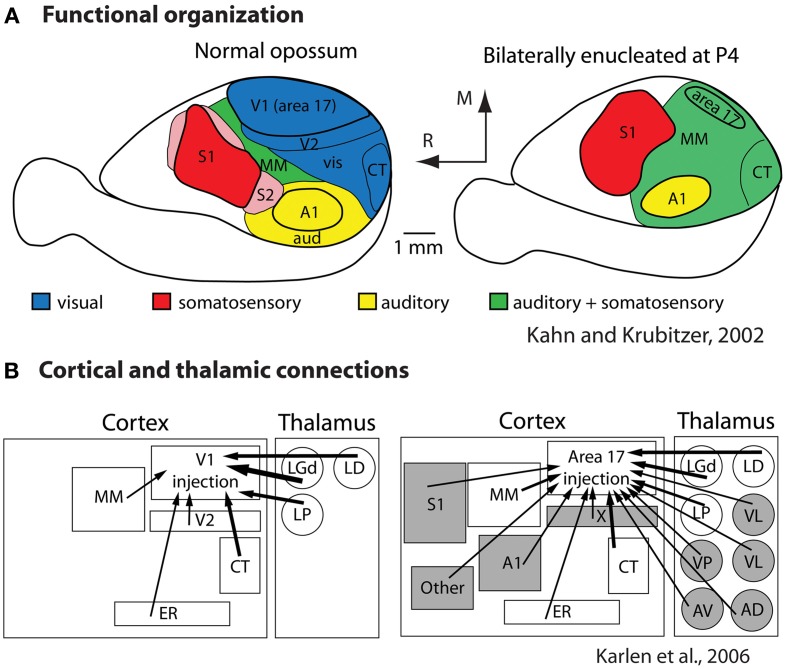
**Alterations in the functional organization (A) and connectivity (B) in bilaterally enucleated opossums.** In normal animals (left) much of cortex is devoted to visual processing. With early and complete loss of vision (right) all of what would normally develop into visual cortex is taken over by the spared sensory systems. This functional reorganization is accompanied by alterations in thalamocortical and corticocortical connections **(B)**. Modified from Kahn and Krubitzer (2002) **(A)**; Karlen et al. (2006) **(B)**. Conventions as in previous figures.

To determine the extent to which sensory receptor arrays, as well as inputs from multiple sensory systems, contribute to aspects of the cortical phenotype, our lab bilaterally enucleated short-tailed opossums very early in development, before thalamocortical afferents reached the cortex and before retinal ganglion cell axons reached the thalamus (Taylor and Guillery, 1994; Molnár et al., 1998). We found that loss of visual input results in a massive reallocation of sensory cortex (cortical domain changes) in that “visual cortex” is functionally taken over by the auditory and somatosensory systems (Kahn and Krubitzer, 2002; Figure 8A). This early loss of visual input resulted in a decrease in the size of architectonically defined V1 as well as an increase in the size of S1 (Karlen and Krubitzer, 2009). Further, cortex in the expected location of V1 received aberrant inputs from somatosensory and auditory structures of the cortex and thalamus (Karlen et al., 2006; Figure 8B). Studies in anophthalmic mice have also demonstrated alterations in subcortical connections and large changes in functional organization of “visual cortex” (Godement et al., 1979; Chabot et al., 2007), and studies of congenitally deaf mice show that auditory cortex is taken over by the visual and somatosensory systems (Hunt et al., 2006). Work in experimentally deafened cats supports these data. Cats that are deafened early have superior peripheral visual localization and motion detection abilities, and these abilities can be abolished when specific areas of auditory cortex are deactivated (the posterior auditory field and the dorsal zone, respectively; Lomber et al., 2010). Recently it was shown that projections from extrastriate visual areas to the dorsal zone of auditory cortex provides the anatomical substrate for this behavioral plasticity (Kok et al., 2013).

Armed with knowledge of the types of alterations that occur with extreme changes in receptor array and major loss of sensory input, scientists can determine if these same types of alterations occur with changes in environmental context in which the animal develops. There are numerous examples in the auditory, visual, and somatosensory systems that demonstrate physical rearing conditions produce changes to the cortical phenotype. For example dark-rearing or stripe-rearing in cats and ferrets leads to a decrease in neural responses to visual stimuli in orientations in which the manipulated animals lack experience (e.g., Blasdel et al., 1977; Sengpiel et al., 1999; Li et al., 2006) and recent studies in rodents have corroborated these results, demonstrating that diverse visual experience is necessary for normal visual development (O'Hashi et al., 2007; Kreile et al., 2011). Similar findings have been found in the auditory system, in which repeated presentation of specific auditory stimuli early in development produces an expansion of the cortical representation of the tones presented (Zhang et al., 2001). Finally, an increase in the amount of cortex devoted to representing particular regions of the body have been generated either through extensive use of the animal's optimal effector (Recanzone et al., 1992), or in some cases, training using the non-optimal effector (Tennant et al., 2012).

**Figure 9:**
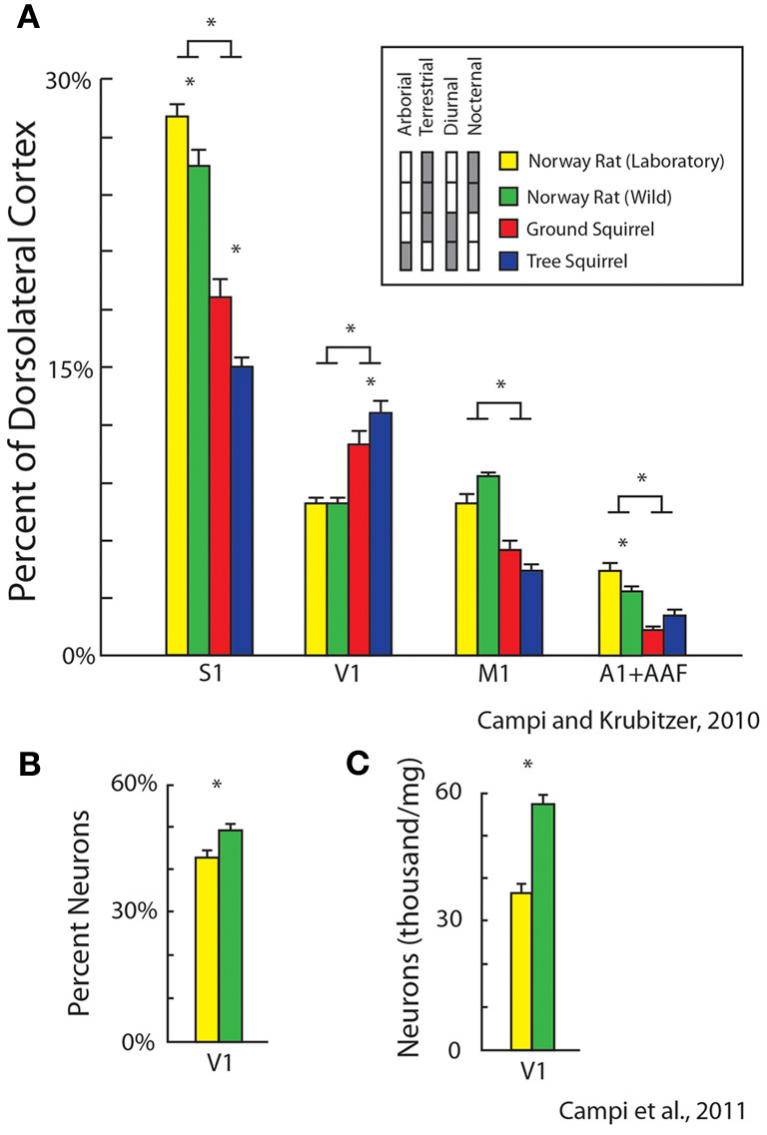
**Alterations in the relative size of primary cortical areas (A) and cellular composition (B,C) between different populations of rodents.** These features of organization are related to lifestyle (diurnal vs. nocturnal; arboreal vs. terrestrial) and rearing condition (laboratory, yellow vs. wild-caught, green). Bars represent mean ± standard error, asterisks represent statistical significance. Modified from Campi and Krubitzer (2010) **(A)**; Campi et al. (2011) **(B,C)**.

Recently we examined the effects of lifestyle and exposure to radically different sensory environments on the size and cellular composition of cortical fields in different rodents for different sensory systems. First, we quantified relative cortical field size in diurnal vs. nocturnal rodents and terrestrial vs. arboreal rodents (Figure 9A). We found differential expansions and contractions of visual, auditory and somatosensory cortex that were related to lifestyle. For example, diurnal squirrels had a relatively larger V1 while nocturnal rats had a relatively larger S1 and A1 (expressed as a percentage of the entire cortical sheet, Campi and Krubitzer, 2010). Furthermore, arboreal squirrels, which live in a visually demanding environment, had a larger V1 and showed an expansion of visual cortex compared to terrestrial squirrels.

We also quantified and compared differences in cortical field size and cellular composition of primary visual cortex between wild-caught Norway rats and Norway rats reared in the laboratory. Obviously the sensory experience, motor demands and sensory mediated behaviors in a natural (and pervasive) environment are more dynamic and complex than the more limited demands of the laboratory environment. We found that there were significant differences in the size of primary sensory areas with laboratory rats having larger a S1 and A1 compared to wild caught animals (Campi and Krubitzer, 2010; Figure 9A). Conversely, V1 in wild caught rats had a larger percentage of neurons and a greater density of neurons compared to laboratory rats (Campi et al., 2011; Figures 9B,C). These studies indicate that the fundamental structure of neocortex can be modified through experience.

### What are the mechanisms that generate epigenetic alterations to the neocortex?

Decades of studies on developmental and adult plasticity of the neocortex demonstrate that sensory experience can profoundly transform features of cortical organization that are known to be altered throughout the course of evolution, such as cortical field size, organization, cellular composition, neural response properties, and connectivity. Rather than a simple and small refinement of parameters initiated by genes, experience plays a critical role in the construction of the neocortex. This should not be surprising since the role of the neocortex appears to be that of a comparative predictor for the generation of adaptive behavior, and behavior is the target of natural selection. These behaviors are often tightly temporally correlated with the stimulus, or temporally uncorrelated in which a substantial amount of time may have elapsed between the stimulus and behavior. In any case, the predictive precision of the neocortex is built by accurate representations of the physical context or collective biomass in which the animal develops and behaves. Thus, it is not surprising that there are mechanisms that allow the animal, and the brain, which generates its behavior, to change substantially within a lifetime.

Although the field of epigenetics has had a recent resurgence, as noted above, the notion that a number of processes occur between the genotype and the ultimate phenotype has been appreciated since the last half of the previous century (Holliday, 2006 for review). The current review has attempted to provide a number of concrete examples in which aspects of the cortical phenotype can vary based on a number of different genetic and experience dependent factors. While describing the details of epigenetic mechanisms is beyond the scope of our laboratory's purview, it would be remiss not to discuss how environmental signals program the operation of the genome, and the mechanisms by which these effects endure beyond the period of exposure during development (Kappeler and Meaney, 2010).

Some of the best examples of these interactions come from studies of mother–offspring interactions in rats. Maternal licking and grooming (LG) of pups is a variable trait in Long-Evans rats (Champagne et al., 2003), and the frequency of the maternal LG is dictated by environmental factors such as stress levels and light/dark cycles (Champagne and Meaney, 2006; Toki et al., 2007). For example, natural variations in LG of pups during the early postnatal period affect the development of the hypothalamic–pituitary–adrenal (HPA) axis (Liu et al., 1997; Caldji et al., 1998; Menard et al., 2004). In adulthood, offspring of high LG mothers have lower circulating adrenocorticotropic hormone levels during stress. This blunted stress response is associated with changes in glucocorticoid receptor (GR) mRNA and protein expression in the hippocampus, which regulates glucocorticoid feedback sensitivity (Weaver et al., 2004). Importantly, these behavioral effects and changes in gene expression that regulate the HPA in adults are initiated by mother-infant interactions during the early postpartum period. It is proposed that during the early postnatal period variations in tactile stimulation during LG induce epigenetic modifications in the promoter region of the GR gene resulting in alterations of GR expression in the hippocampus that persist throughout life. Increased tactile stimulation received by offspring of high LG mothers results in an increase in neurotransmitter binding and subsequent intracellular signaling in the hippocampus, which activates GR gene transcription. Importantly, the pattern of increased GR transcription persists into adulthood because of a reduction in methylation of the GR gene. DNA methylation is typically associated with a repression of gene expression (Miranda and Jones, 2007; see Kappeler and Meaney, 2010 for review), therefore a reduction in methylation of the promoter region of the GR gene is associated with increased GR gene expression (Meaney and Szyf, 2005). This modification of the genome and the behaviors ultimately generated by these changes can be transmitted to the second generation offspring, but are reversed with cross-fostering (rearing low LG pups with high LG parents).

There are also examples of epigenetic mechanisms operating directly on the nervous system. Work by Putignano et al. (2007) demonstrates that during visual critical periods, sensory inputs can directly turn on and off regulatory factors which alter the accessibility of gene promoters. When these genes are made experimentally accessible in adulthood, much of the ocular dominance plasticity that is observed early in development is reinstated (Putignano et al., 2007). Further studies have identified a specific histone deacetylase (HDAC9) which has been shown to translocate from the nucleus to the cytoplasm following neural activity early in development. When HDAC9 was experimentally prevented from translocating, manipulated cells showed decreased dendritic branches, while knockdown of HDAC9 increased dendritic growth (Sugo et al., 2010).

Despite the constraints imposed by genes and the contingencies of genetic cascades, and the laws of physics that govern all forms of matter and energy, biological organisms have evolved mechanisms that allow them to loosen these constraints and dynamically adapt both within a lifetime and across generations. In a sense, the strength of this evolvability (Earl and Deem, 2004) and the evolution of a large, malleable comparative predictor (neocortex), rather than specific genes or gene products, may be one of the fundamental differences that distinguish humans from other animals.

### Conflict of interest statement

The authors declare that the research was conducted in the absence of any commercial or financial relationships that could be construed as a potential conflict of interest.
